# JC Virus Seroprevalence and JCVAb Index in Polish Multiple Sclerosis Treatment-Naïve Patients

**DOI:** 10.3390/jcm9123867

**Published:** 2020-11-27

**Authors:** Robert Bonek, Wojciech Guenter, Robert Jałowiński, Anna Karbicka, Anna Litwin, Maciej Maciejowski, Radosław Zajdel, Veronique Petit, Konrad Rejdak

**Affiliations:** 1 Department of Neurology and Clinical Neuroimmunology, Regional Specialist Hospital, 86-300 Grudziadz, Poland; 2 Foundation Supporting Development of Neurology and Clinical Neuroimmunology MoA, 85-654 Bydgoszcz, Poland; 3 Department of Clinical Neuropsychology, Nicolaus Copernicus University, 87-100 Torun, Poland, and Collegium Medicum, 85-094 Bydgoszcz, Poland; wojciech.guenter@gmail.com; 4 Department of Neurology, Regional Hospital, 71-455 Szczecin, Poland; rojal@esculap.pl (R.J.); anuszek-4@o2.pl (A.K.); 5 Department of Neurology, Regional Hospital, 10-561 Olsztyn, Poland; annalitw@gmail.com; 6 KMK Clinical, MS Center, 40-571 Katowice, Poland; maciejmaciejowski@poczta.onet.pl; 7 Chair of Business Informatics, University of Lodz, 90-214 Lodz, Poland; radoslaw.zajdel@uni.lodz.pl; 8 Department of Neurology, Medical University of Lublin, 20-090 Lublin, Poland; vpetit@onet.pl (V.P.); konradrejdak@umlub.pl (K.R.)

**Keywords:** multiple sclerosis, John Cunningham virus, anti-JCV antibody, antibody index, seroprevalence

## Abstract

Multiple sclerosis (MS) treatment with new agents is associated with the risk of the development of progressive multifocal leukoencephalopathy (PML). The seropositivity and a high index of anti-John Cunningham virus (JCV) antibodies are some of the risk factors for PML development. The aim of this study was to assess the seroprevalence of anti-JCVAb and JCVAb index (AI), as well as its correlations with demographic and clinical characteristics in treatment-naïve Polish MS patients. This is a multicenter, prospective, and cross-sectional study involving 665 MS patients. The overall prevalence of anti-JCVAb was 65.3%, while 63.1% of seropositive patients had an index level of >1.5. The seroprevalence was shown to increase along with the patient’s age. Except for age, the prevalence of anti-JCVAb was not associated with demographic or clinical data. No correlations between the index levels and the demographic or clinical data were observed. In Poland, the seroprevalence of anti-JCVAb in treatment-naïve MS patients is one of the highest in Europe. The majority of seropositive patients had an anti-JCV antibody level denoting a high-risk category. This means that we need further studies to be conducted on the individualization of MS treatment in order to provide patients with an appropriate therapeutic safety level.

## 1. Introduction

The John Cunningham virus (JCV/JCPyV) is an omnipresent and species-specific, human DNA virus belonging to the *Polyomaviridae* family. In the majority of cases, exposure to JCV occurs in childhood without causing any clinical manifestation. After the primary infection, the JCV remains in a latent form in the kidney cells, bone marrow, and lymphoid tissue for many years. Depending on the country, the JCV seropositivity rate in the general population ranges from 33% to 91% [1,2].

JCV is an etiological agent of progressive multifocal leukoencephalopathy (PML). This is a rare, but frequently fatal, demyelinating disease of the central nervous system (CNS), during which lytic oligodendrocyte injuries are observed. The first cases of PML were reported in 1958 in three patients with chronic lymphocytic leukemia (CLL) and Hodgkin’s disease. In 1971, the JCV was isolated from the brain of a Hodgkin’s disease patient as the first of the 13 currently known polyomaviruses [3,4,5].

PML develops as a reactivation of the latent neurotropic virus strain infection in immunosuppression conditions associated with the lack of immune—especially T-cell-dependent—supervision. Initially, regardless of the treatment used, PML was considered as a rare complication of hematological cancers or autoimmune diseases, such as systemic lupus erythematosus (SLE) or sarcoidosis. A substantial increase in PML rates was observed during the acquired immune deficiency syndrome (AIDS) pandemic when up to 5% of human immunodeficiency virus (HIV)-positive patients developed this condition. PML cases were also observed in patients undergoing solid-organ and stem-cell transplantation [1,6,7].

The advances in medical treatment in recent years demonstrated that the occurrence of PML cases is currently considered as a complication of treatment with biological agents, initially mainly monoclonal antibodies (mAbs), such as natalizumab, efalizumab, rituximab, or infliximab, of autoimmune diseases such as multiple sclerosis (MS), psoriasis, Crohn’s disease, or SLE [8].

In this situation, the occurrence of PML as a complication of MS treatment seems to be of extreme importance because, after many years of therapeutic nihilism, we finally have several disease-modifying therapies (DMTs) which allowed for substantial therapeutic advances. However, the use of more active agents is also associated with an increase in PML cases considered as a treatment complication. The association between natalizumab therapy and PML development in MS significantly increased the interest in the issue mentioned above. Regarding that, a stratification strategy of MS patients treated with natalizumab (NTZ) was developed to improve their safety and to limit the risk of PML occurrence. The risk factors for PML development in patients treated with natalizumab involve the JCV seropositivity, the previous immunosuppressive treatment, and NTZ treatment duration of more than 2 years. The JCV seropositivity is evaluated with a two-step enzyme-linked immunosorbent assay (ELISA) detecting anti-JCV antibodies (JCVAb) in blood serum. In seropositive persons, further risk stratification is possible by using categories of anti-JCV antibody index (AI) levels considering 0.9 and 1.5 as cutoff values. Taking that into account, three risk categories—low (up to 0.9), intermediate (from 0.9 to 1.5), and high (more than 1.5)—were distinguished [9].

The majority of the previous data on the JCV serostatus and antibody index levels come from trials involving patients treated with natalizumab and conducted in Western Europe and North America. It was stated that approximately 50–60% of patients were JCV-positive, and up to 45% had AI levels > 1.5 [10,11,12].

Reports on the PML cases in patients receiving other DMTs, such as fingolimod, dimethyl fumarate, and ocrelizumab, increased the importance of the PML issue considered as a complication of MS treatment [8,13]. Taking that into account, it seems that every patient should have a JCV serostatus and index level measured before treatment initiation.

In search of other factors affecting the JCVAb prevalence, some previous studies also evaluated the correlations between the specific clinical and demographic data measured in study populations. It was stated that the JCV seropositivity was associated with older age and the male sex and differed between countries but without any specific geographic pattern [11,14,15,16,17,18,19,20,21,22].

So far, there has been no study evaluating the JCV serostatus and antibody index levels in the Polish population of patients with multiple sclerosis (PwMS). Therefore, this study aimed to assess the JCVAb serostatus and index in MS patients and eventual associations between the serostatus/index and clinical and demographic factors. The study was performed only in treatment-naïve patients to exclude an eventual effect of the previous and ongoing therapy.

## 2. Materials and Methods

### 2.1. Patients

This was a multicenter, prospective, and cross-sectional study involving five MS centers located in different regions of Poland (Bydgoszcz/Grudziądz, Katowice, Lublin, Olsztyn, and Szczecin), conducted from July 2014 to January 2018. Overall, 665 treatment-naïve patients with multiple sclerosis (MS) diagnosed according to the 2010 modified McDonald’s criteria were enrolled [23].

All the patients were above 18 years old. The following demographic and clinical data were collected: age, sex, disease duration, disability level measured as the Expanded Disability Status Scale (EDSS) score, and the MS course.

The exclusion criteria involved those who were under 18 years old, therapy with corticosteroids within 4 weeks of serum sampling for JCVAb level measurement, and the use of intravenous immunoglobulins in the previous 6 months.

The primary endpoint was the prevalence of JCVAb in treatment-naïve MS patients and the associations between the presence of JCVAb and the collected demographic and clinical data. The secondary endpoints involved JCVAb index level measurement, as well as the associations between the antibody index (AI) level categories and demographic and clinical data.

The Ethics Committee at the Regional Chamber of Physicians and Dentists in Bydgoszcz, Poland—the ethics committee of the coordinator center (No. 29/2014)—approved the study. All of the patients provided a signed informed consent form.

### 2.2. Samples

All the samples tested for anti-JCV antibody serostatus and index were analyzed by the reference laboratory (UNILABS) in Copenhagen (Denmark). The second-generation confirmatory ELISA (STRATIFY JCV™ DxSelect—STRAFITY2) test was used for testing the sera for anti-JCV antibodies and index levels. The testing procedure consisted of a screening enzyme-linked immunosorbent assay (ELISA) and a (supplemental) confirmatory test. In a screen-testing procedure, anti-JCV antibodies index levels of <0.2 and >0.4 were considered as negative and positive, respectively. Samples with index levels between 0.2 and 0.4 underwent a confirmatory test (second step), in which the results of >45% were classified as an anti-JCV antibody (JCVAb)-positive [24].

### 2.3. Statistical Analysis

The results were analyzed with the use of statistical methods, including some multidimensional tests. The Shapiro–Wilk test was used to assess normal distribution. The studied characteristics with non-normal distribution, as well as qualitative and quantitative data, were analyzed with the use of nonparametric tests, including the Kruskal–Wallis ANOVA, Pearson’s chi-squared, and Mann–Whitney U tests. General descriptive statistics methods were also used. A logistic regression model was used for multivariate analysis, where applicable. The statistically significant *p* level was set at <0.05.

## 3. Results

### 3.1. Patients

The study enrolled 665 treatment-naïve MS patients from five Polish MS centers. Table 1 shows the participants’ clinical and demographic data. Overall, 68.7% of the patients were females; the mean age in the entire study cohort was 42.7 ± 12.7 years (range, from 18 to 78 years). In total, the study involved 91.1% of patients below 60 years old. The most common clinical MS phenotype was relapsing–remitting multiple sclerosis (RRMS), which was diagnosed in 66.3% of the patients. The mean MS duration from the onset of the first symptoms was 8.2 ± 8.8 years (range, from 0 to 50 years). The median disability score measured with the EDSS scale was 3.0 (range, 1.0 to 9.0).

### 3.2. Prevalence and Index of Anti-JCV Antibodies

Of the 665 enrolled patients, 434 had anti-JCV antibodies, resulting in an overall anti-JCVAb prevalence index of 65.3%. There were no significant differences in the seroprevalence of anti-JCVAb depending on the patient’s sex (65.9% in males and 65% in females), disability level assessed by the EDSS score, and disease course. Although an MS course analysis revealed the lowest in RRMS (64.3%) and the highest anti-JCV Ab levels in primary progressive multiple sclerosis (PPMS) patients (72.3%), the observed difference was not significant (*p* > 0.05; Table 2).

After an additional request, we obtained the results of anti-JCVAb index levels in all patients, both seropositive and seronegative, enrolled in the study. In our treatment-naïve MS patients, the mean Abs index (AI) level for the entire cohort amounted to 1.44 ± 1.30 (median 0.93); the lowest and the highest levels were 0.03 and 4.36, respectively. In the seropositive group, the mean AI level was 2.11 ± 1.15 (median 2.22), while the range amounted to 0.25–4.36. In the seronegative group, the parameters mentioned above were as follows: AI 0.20 ± 0.07 (median 0.18), range 0.03–0.40. No significant statistical differences in AI by the patient’s sex, disability level, and clinical disease phenotype were observed. However, the RRMS patients had the lowest (1.41), while the PPMS patients had the highest mean AI levels (1.63) (*p* > 0.05; Table 2).

In addition, our results did not demonstrate any differences in the seroprevalence of anti-JCVAb, as well as antibody index (AI) values, depending on the disease duration measured from the date of onset of the first clinical symptoms (Table 3).

**Figure 1:**
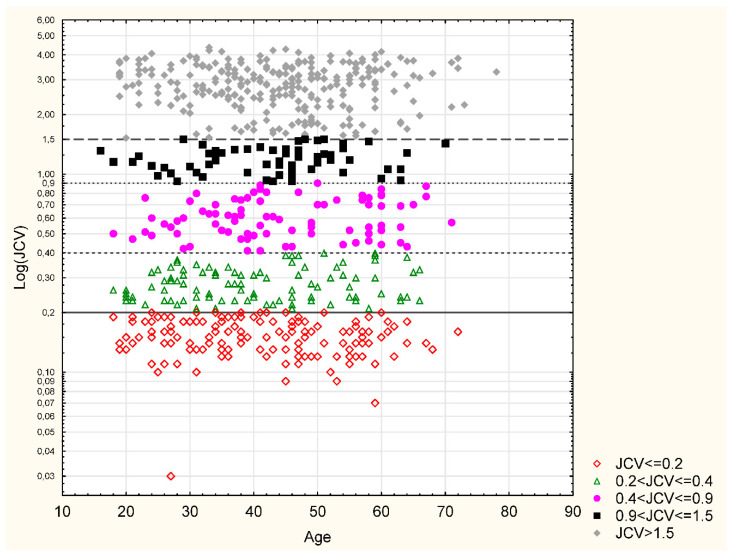
Anti-JCV antibody index (AI) categories and age.

The analysis of the patient’s age at the time of drawing the sample for anti-JCVAb measurement revealed an increase in the seroprevalence of anti-JCVAb over time, along with the MS patient’s age. Furthermore, the difference between the youngest and other groups of patients was significant (*p* = 0.007). The seroprevalence stratified by quintiles was as follows: 51.3% in 18–29-year-olds, 64% in 30–39-year-olds, 69.5% in 40–49-year-olds, 70.2% in 50–59-year-olds, and 71.2% in the >60-year-olds. The lowest mean anti-JCVAb index (AI) was observed in the youngest age group (1.19 ± 1.30) and was shown to increase over time up to 60 years old (1.5 ± 1.30), but the difference between age groups was not significant (Table 4, Figure 1).

Table 5 shows anti-JCVAb index categories for the entire cohort. In a group of 434 seropositive patients, 10 (2.3%) persons had an AI between 0.2 and 0.4, 87 (20.1%) had an AI from 0.4 to 0.9, 63 (14.5%) from 0.9 to 1.5, and 274 (63.1%) patients achieved an AI of >1.5. No differences regarding the AI category by sex, disease type, EDSS score, age at disease onset, and disease duration were observed (Table 5 and Table 6). Figure 1 shows the distribution of AI values by categories depending on the age at the time of drawing the sample.

Regardless of the descriptive statistics given in the Table 2, Table 3 and Table 4 (presenting data in stratified way), we performed the nonparametrical *r* Spearman analysis, as well as ANCOVA with “age” as a covariant. Results revealed that correlation between EDSS score and antibody index was statistically significant (*p* = 0.0296); however, the *r*-value was very low (0.0846), which meant that correlation was very weak (clinical significance was unlikely). ANCOVA (age as a covariant) showed no statistical significance (*p* = 0.4852) regarding interdependence of EDSS and antibody index with age to be irrelevant modifying factor (*p* = 0.6730). The presented statistical pipeline was run again with disease duration and antibody index. Correlation was shown to be nonsignificant (*p* = 0.5480). ANCOVA confirmed the earlier findings—there was no interdependence (*p* = 0.2188) and age had no modifying impact (*p* = 0.1172).

## 4. Discussion

This is the first multicenter study on the seroprevalence of JCVAb in the Polish MS population. The unique aspect of the study is the fact that it was conducted in the treatment-naïve MS patients, which allowed us to exclude the effect of MS therapies on the JCVAb seroprevalence or index levels. The reason for this is the previous inconsistent data from the PwMS population, demonstrating the relation of the prevalence of JCVAb and index levels during the sampling to drug-modifying therapy (DMT) used. On the one hand, we have data confirming no link between the immunomodulatory or immunosuppressive treatment used and JCVAb seroprevalence [10,11,14], while, on the other hand, the available data demonstrate that the use of immunosuppressive agents is associated with the higher seroprevalence of JCVAb in study groups [18]. In addition, it was also found that the duration of natalizumab exposure was a risk factor for the higher prevalence of JCVAb potentially resulting from an asymptomatic JC virus reactivation [15], while the treatment time correlated with an antibody index (AI) increase [25]. The previous observations also revealed that the initiation of treatment with natalizumab was associated with a decrease in AI level, potentially suggesting the immunosuppressive effect of this agent. The mechanism of action (MoA) responsible for such an effect is not fully known, but the observed difference in AI levels was not significant [17,21]. A recent study evaluating rituximab, a chimeric monoclonal anti-CD antibody, showed a significant effect of the treatment on the decrease in the JCVAb index in 76% of seropositive patients with a mean AI decrease of 14%. Moreover, the majority of patients presented a JCVAb level decrease over time. Furthermore, that study also demonstrated a slight decrease in the seropositivity rate from 76% to 71% in the study cohort [26]. The effect of rituximab on the AI level mentioned hereinabove was confirmed in another study, which also revealed a significant decrease in AI levels during fingolimod treatment [27].

An increasing interest in JCV prevalence in MS patients was observed along with an introduction of natalizumab, a breakthrough treatment of relapsing–remitting MS (RRMS). However, in some patients, that therapy caused severe complications, such as progressive multifocal leukoencephalopathy (PML). That situation intensified the studies conducted on the stratification of risk for the development of PML [8,12]. Currently, risk factors for developing PML include the presence of JCVAb, previous immunosuppressive agent use, and duration of natalizumab therapy of more than two years, as well as an AI level >1.5 [9]. In recent years, the issue of PML development after MS treatment took on increased clinical importance when several disease-modifying therapies (DMTs) were introduced. It was revealed that the use of some of them is rare but still associated with PML development. Such a link was observed for fingolimod, dimethyl fumarate, and, more recently, ocrelizumab, which is a humanized anti-CD20 monoclonal Ab [1,13]. We also cannot forget that other agents are still in development, and their effect on the JCV seroprevalence and eventual PML risk is not fully known. Therefore, regardless of treatment, the identification of associations between the demographic and clinical factors and the prevalence of JCV in the MS population and Ab index levels is of such importance. Therefore, the abovementioned data enables the better optimization of treatment selection to obtain an appropriate balance between the efficacy and safety of therapy, which in this case is the risk of PML development.

The overall prevalence of JCVAb in the Polish treatment-naïve MS patients amounted to 65.3%, which was higher compared to the overall seroprevalence observed in two previous international studies demonstrating a JCV seropositivity rate of 57.1% and 57.6% [11,19]. In addition, we noted that the abovementioned level is one of the highest observed compared to the previous studies using the STRATIFY JCV test to assess the JCV prevalence in MS patients. That allows classifying Poland as a country with a high JCV seropositivity rate. Compared to the results of the JEMS trial and previous studies using the STRATIFY JCV test to evaluate JCVAb levels, Poland would be ranked fifth after Portugal (69.5%), Turkey (67.7%), the Netherlands (66.7%), and Austria (66.7% and 66.2%), but before Germany (61% and 59.1%), Sweden (59.0%), Italy (58.3%), France (57.6%), Israel (56.6%), Canada (56.3%), Switzerland (55.6%), Belgium (54.4%), Spain (53.5%), Denmark (52.6%), Ireland (51%), the United Kingdom (48.8%), Australia (48.6%), Norway (47.4%), and Cyprus (45.8) [11,19,22,28]. Of course, we also have to mention that such a comparison is limited by some small national patient cohorts included in the study. Another limitation of the JEMS and many previous trials is the fact that they used the STRATIFY JCV test, while our study was based on the results obtained with the STRATIFY JCV DxSelect test. In contrast to the first-generation test, the latter method exhibits an increased capacity to detect JCV Abs at a low response level and higher reproducibility, as highlighted by a recent study. Naturally, the majority of results obtained with the use of the first- and second-generation test are consistent [19,29,30].

Regarding studies performed in other European countries with the use of the STRATIFY JCV DxSelect test, our results showed the highest seropositivity rate in Polish MS patients compared to Portugal (60.8%), Spain (60.5%), Czech Republic (59%), United Kingdom (59%), Finland (57.4%), Austria (52.3%), or France (49.7%) [21,22,25,30,31,32,33]. Concerning other regions of the world, the prevalence of JCVAb in our cohort is higher compared to the Eastern Mediterranean region, where the presence of anti-JCV antibodies was noted in 48.7% of patients [34]. In contrast, the results of the recent study on the JCV Abs seroprevalence in the Iranian cohort demonstrated higher levels (67.9%) compared to our group [35]. However, data from the Eastern Mediterranean region, similarly to South America where the Brazilian data demonstrated a positive JCV serostatus in 57.1% of patients [36], are compatible with the data obtained in Europe, Canada, and Australia. A substantial difference can be seen in populations of patients from East Asia, where the prevalence of JC virus amounted to 69.5% in Japan and 80% in China and South Korea [37,38,39]. The reason for such a big difference in the JCV seropositivity rate between East Asia and other regions of the world remains unclear.

The higher prevalence of JCVAb in Poland compared to other European countries may result from the patient selection. The other cohorts, entirely (Czech Republic) or partially, consisted of patients treated with natalizumab, with the previous immunomodulatory or immunosuppressive therapy [21,25,30,31,33].

The previous studies demonstrated the link between an increase in the JCVAb seropositivity rate and older age and the male sex [11,14,16,17,19,21]. Our study supports the previous data demonstrating that the JCV seropositivity rate is associated with the older age. This confirms the hypothesis of the continuous increase of the infection rate with age, with the highest level in the oldest age groups (more than 70% of the population above 50 years old is infected). The prevalence mentioned above is higher compared to the JEMS trial and the second international cohort, as well as in the Spanish study, but lower than the Finnish study where almost 80% of the population over 60 years old was infected, although the sample size was small [19,21,22]. Our data do not confirm the initial information about the association between the higher anti-JCV antibody prevalence and male sex. This is in line with the data obtained in recent years in France, Spain, Portugal, and South Korea. However, despite the trend observed in those countries, the difference was not significant [20,22,29,30,31,38].

The data from our study are according to the previous data demonstrating no relation of disease duration, disease course, and the disability level measured on the EDSS scale to the seroprevalence [19,21,30].

The mean JCVAb index (AI) in total (1.44) and seropositive patient (2.11) populations is similar to the results of the Czech cohort, in which AI levels were 1.29 and 2.09, respectively. Moreover, our results are comparable with data from Portugal and Iran, where the mean AI in seropositive patient groups was 2.1 and 2.23, respectively [30,33,35]. When comparing the median AI in seropositive patients, our levels (median of 2.22) are comparable with the data obtained in Austria (2.3) and higher than in Finland (1.64) [21,40]. Similarly to the JCV seroprevalence, the highest levels of the antibody index were observed in East Asian countries; the median levels observed in China and South Korea were 3.17 and 3.27, respectively [38,39].

In the seropositive group, similarly to the previous studies, the lowest (2.3%) population of patients had an AI within indefinite levels, i.e., from ≥0.2 to ≤0.4, which was confirmed positive on the subsequent measurement [30].

The analysis of the index category prevalence performed in our study demonstrated that the group with AI levels > 1.5 was the most prevalent, being 41.2% of the entire MS patient cohort. This means that our high-risk group is lower compared to the Portuguese group, in which the latest JCVAb index >1.5 consisted of 45.5% of immunosuppressant-naïve patients and the Austrian group (45% of patients) but higher than in other European countries: Germany 22%, France 33%, the United Kingdom 33%, or Spain 39.9%. In total, 61.3% of seropositive patients had AI >1.5; similar, slightly higher results were observed in Portugal (63.7%) [22,25,30,40,41]. The highest prevalence of the high-risk AI category, similarly to the seroprevalence and index level, was observed in China, South Korea, and Japan (78%, 77%, and 75%, respectively) [37,38,39].

The presence of such a numerous group of patients with an AI >1.5 has significant clinical significance for treatment planning. As previously stated, the AI level does not significantly impact the risk of PML development in immunosuppression-naïve seropositive patients receiving natalizumab for the first 2 years. However, after 2 years of treatment, that risk increases considerably, especially in the AI >1.5 category [9]. This became even more important with the introduction of anti-CD20 monoclonal antibodies (mAbs) into the MS treatment. We have to remember that in addition to the previous studies evaluating the effect of rituximab on the AI levels. Moreover, we should keep in mind the reports of rituximab-induced PML cases in patients with autoimmune diseases or cancer [40]. Regarding that, we should determine the JCVAb seroprevalence and index levels during the use of new anti-CD20 mAbs, such as ocrelizumab or ofatumumab, especially in patients previously receiving immunosuppressive treatment.

We conducted an analysis of the data obtained from the Polish MS patients who did not show any correlation between the antibody index and any demographic or clinical parameter, which is in line with the Spanish multicenter data [29]. Of course, we observed an increase in the index levels along with the patient’s age, but the difference, similarly to German cohort, was not significant. In contrast, the Czech and Austrian data showed a different outcome [33,41,42].

Our study had some limitations. First of all, a limited group selection concerning only treatment-naïve patients reduced the number of enrolled participants. Secondly, the study lacked longitudinal data.

## 5. Conclusions

Our study demonstrated that the seroprevalence of JCVAb in Polish MS patients is one of the highest in Europe, amounting to 65.3%. The study only concerned treatment-naïve patients. Thus, the eventual skewing of the results, especially concerning the AI levels, from the treatment used, was eliminated. Therefore, the presence of a high anti-JCV antibody index of >1.5 in 61.3% of seropositive patients is an essential factor indicating the need for individualized therapy selection from the disease onset. Regarding the previously described correlations, our study confirmed the significant association between the patient’s age and the seroprevalence rate. The reasons for the occurrence of high levels of the JCVAb index and their prevalence in the Polish population require further studies.

## Figures and Tables

**Table 1:** Clinical and demographic characteristic of multiple sclerosis (MS) patients at the time of the STRATIFY John Cunningham virus (JCV) DxSelect test.

Characteristic	Study Population
Number of patients, *n*	665
Sex, female/male, *n*	457/208
Mean age, years ± SD	42.7 ± 12.7
Age, median (range)	43 (18–78)
Age categories, *n* (%)	
18–29 years	117 (17.6)
30–39 years	161 (24.2)
40–49 years	187 (28.1)
50–59 years	134 (20.2)
≥60 years	66 (9.9)
Disease course, *n* (%)	
RRMS	441 (66.3)
SPMS	107 (16.1)
PPMS	117 (17.6)
Disease duration, median (range)	5.0 (0–50)
Disease duration categories, *n* (%)	
0–5 years	361 (54.3)
6–10 years	112 (16.8)
11–15 years	66 (9.9)
≥16 years	126 (19.0)
EDSS, median (range)	3.0 (1.0–9.0)

RRMS—relapsing–remitting multiple sclerosis, SPMS—secondary progressive multiple sclerosis, PPMS—primary progressive multiple sclerosis, EDSS—Expanded Disability Status Scale, SD—standard deviation.

**Table 2:** Anti-JCV antibody prevalence and antibody index by the patients’ sex, disease course and EDSS score.

*n* (%)	All	F	M	MW-U	RRMS	SPMS	PPMS	KW-H	EDSS I	EDSS II	EDSS III	KW-H
**JCV+**	434 (65.3)	297 (65.0)	137 (65.9)	*p* > 0.05	279 (63.3)	70 (65.4)	85 (72.6)	*p* > 0.05	150 (59.8)	173 (68.9)	111 (68.1)	*p* = 0.07
**JCV−**	231 (34.7)	160 (35.0)	71 (34.1)		162 (36.7)	37 (34.6)	32 (27.4)		101 (40.2)	78(31.1)	52 (31.9)	
**Mean**	1.44	1.43	1.47	*p* > 0.05	1.41	1.39	1.63	*p* > 0.05	1.32	1.52	1.51	*p* > 0.05
**Median**	0.93	0.97	0.88		0.81	0.78	1.34		0.61	1.17	1.06	
**Min**	0.03	0.03	0.09		0.03	0.09	0.11		0.10	0.03	0.07	
**Max**	4.36	4.36	4.06		4.36	4.01	4.07		4.36	4.21	4.01	
**SD**	1.30	1.29	1.32		1.29	1.31	1.31		1.29	1.30	1.29	

F—female, M—male, RRMS—relapsing–remitting multiple sclerosis, SPMS—secondary progressive multiple sclerosis, PPMS—primary progressive multiple sclerosis, EDSS I—from 0 to 2.0, EDSS II—from 2.5 to 4.5, EDSS III—from 5.0 to 9.0, SD—standard deviation, MW—Mann–Whitney, KW—Kruskal–Wallis.

**Table 3:** Anti-JCV antibody prevalence and antibody index by disease duration (years).

*n* (%)	0–5	6–10	11–15	≥16	KW-H
**JCV+**	225 (62.3)	79 (70.5)	43 (65.2)	87 (69.1)	*p* > 0.05
**JCV−**	136 (37.7)	33 (29.5)	23 (34.8)	39 (30.9)	
**Mean**	1.42	1.55	1.18	1.57	*p* > 0.05
**Median**	0.87	1.19	0.60	1.22	
**Min**	0.07	0.03	0.09	0.11	
**Max**	4.27	4.36	3.96	4.21	
**SD**	1.31	1.32	1.15	1.32	

**Table 4:** Anti-JCV antibody prevalence and antibody index by the patients’ age (years).

*n* (%)	18–29	30–39	40–49	50–59	≥60	KW-H
**JCV+**	60 (51.3)	103 (64.0)	130 (69.5)	94 (70.2)	47 (71.2)	*p* = 0.007
**JCV−**	57 (48.7)	58 (36.0)	57 (30.5)	40 (29.8)	19 (28.8)	
**MW-U**	Corrected Z *p*	*p* > 0.05	*p* > 0.05	*p* > 0.05	*p* > 0.05	
	18–29–30–39 *p* = 0.03					
	18–29–40–49 *p* = 0.001					
	18–29–50–59 *p* = 0.002					
	18–29–≥60 *p* = 0.009					
**Mean**	1.19	1.47	1.52	1.55	1.41	*p* > 0.05
**Median**	0.37	0.80	1.22	1.25	0.78	
**Min**	0.03	0.10	0.09	0.07	0.12	
**Max**	4.06	4.36	4.27	4.07	4.15	
**SD**	1.30	1.33	1.25	1.30	1.32	

**Table 5:** Anti-JCV antibodies index categories and sex, clinical course, and EDSS score.

*n* (%)	All	F	M	RRMS	SPMS	PPMS	EDSS I	EDSS II	EDSS III
JCV ≤0.2	142 (21.35)	99 (21.66)	43 (20.67)	94 (21.32)	26 (24.30)	22 (18.80)	58 (23.11)	51 (20.32)	33 (20.25)
0.2 < JCV ≤ 0.4	99 (14.89)	69 (15.10)	30 (14.42)	73 (16.55)	14 (13.08)	12 (10.26)	47 (18.73)	31 (12.35)	21 (12.88)
		**MW-U *p* > 0.05**		**KW-H *p* > 0.05**			**KW-H *p* > 0.05**		
0.4 < JCV ≤ 0.9	87 (13.09)	56 (12.25)	31 (14.90)	57 (12.92)	16 (14.94)	14 (11.96)	33 (13.15)	30 (11.95)	24 (14.72)
0.9 < JCV ≤ 1.5	63 (9.47)	45 (9.85)	18 (8.66)	41 (9.30)	10 (9.35)	12 (10.26)	22 (8.76)	27 (10.76)	14 (8.59)
JCV > 1.5	274 (41.20)	188 (41.14)	86 (41.35)	176 (39.91)	41 (38.33)	57 (48.72)	91 (36.25)	112 (44.62)	71 (43.56)
		**MW-U *p* > 0.05**		**KW-H *p* > 0.05**			**KW-H *p* > 0.05**		

F—female, M—male, RRMS—relapsing–remitting multiple sclerosis, SPMS—secondary progressive multiple sclerosis, PPMS—primary progressive multiple sclerosis, EDSS I—from 0 to 2.0, EDSS II—from 2.5 to 4.5, EDSS III—from 5.0 to 9.0.

**Table 6:** Anti-JCV antibodies index categories and patients’ age and disease duration.

n (%)	18–29	30–39	40–49	50–59	≥60	0–5		6–10	11–15	>15
JCV ≤0.2	33 (28.21)	35 (21.74)	34 (18.18)	28 (20.90)	12 (18.18)	81 (22.44)	24 (21.43)	10 (15.15)	27 (21.43)
0.2 < JCV ≤ 0.4	26 (22.22)	24 (14.91)	24 (12.83)	17 (12.69)	8 (12.12)	59 (16.34)	10 (8.93)	15 (22.73)	15 (11.90)
**KW-H**	***p* > 0.05**					***p* > 0.05**			
0.4 < JCV ≤ 0.9	12 (10.25)	22 (13.66)	22 (11.77)	15 (11.19)	16 (24.25)	41 (11.36)	15 (13.39)	16 (24.24)	15 (11.90)
0.9 < JCV ≤ 1.5	10 (8.55)	13 (8.08)	21 (11.23)	13 (9.70)	6 (9.09)	38 (10.52)	11 (9.82)	4 (6.06)	10 (7.94)
JCV >1.5	36 (30.77)	67 (41.61)	86 (45.99)	61 (45.52)	24 (36.36)	142(39.34)	52 (46.43)	21 (31.82)	59 (46.83)
**KW-H**	***p* > 0.05**					***p* > 0.05**			
